# Open-Angle and Steroid-Induced Glaucoma in Patients With Retinitis Pigmentosa: A Dangerous Liaison

**DOI:** 10.7759/cureus.55632

**Published:** 2024-03-06

**Authors:** Gabriel A Jiménez-Berríos, Sebastián J Vázquez-Folch, Natalio Izquierdo

**Affiliations:** 1 School of Medicine, Universidad Central del Caribe, Bayamón, PRI; 2 Department of Surgery, School of Medicine, Medical Sciences Campus, University of Puerto Rico, San Juan, PRI

**Keywords:** schwartz-matsuo syndrome, steroid-induced glaucoma, cataracts, retinitis pigmentosa, macular edema

## Abstract

Previous studies have reported that patients with retinitis pigmentosa (RP) may develop open-angle and angle-closure glaucoma.

We conducted a chart review of patients with RP. Two siblings with RP associated with a mutation in the *PDE6B *gene (c. 1540del, p.Leu514Trpfs*61) developed cystoid macular edema (CME) as part of the disease. For this reason, they both underwent intravitreal steroid injections. Both brothers developed steroid-induced glaucoma (SIG). Despite undergoing maximal medical therapy, they underwent seton implants to control their intraocular pressure. A third female patient with RP due to a mutation in the *RPGR *gene underwent cataract surgery. Topical steroids were prescribed and developed SIG.

Increased intraocular pressure remains a complication of topical, injected, and systemic steroids. However, steroids may be needed to treat post-operatively and patients with CME. This case series unveils a complex association between RP and key comorbidities in these patients, with a focus on cataracts, glaucoma, and macular edema. Cataract surgery in patients with RP shows a link to the emergence of glaucoma, particularly in those with *RPGR* and *PDE6B* gene mutations, revealing a novel association with *PDE6B* mutations not previously documented. Furthermore, the paper explores a unique parallel with Schwartz-Matsuo syndrome, suggesting that patients with RP undergoing cataract surgery may develop increased intraocular pressure due to an outflow disturbance akin to Schwartz syndrome. This novel perspective deepens our understanding of the pathophysiological mechanisms governing intraocular pressure dynamics in patients with RP.

To our knowledge, this is the first report of steroid-induced glaucoma in patients with RP due to mutations in the *PDE6B *gene*.* Intraocular pressure evaluation remains of utmost importance in the follow-up of patients with the disease.

## Introduction

Retinitis pigmentosa (RP) is a genetically heterogeneous retinopathy characterized by progressive degeneration and dysfunction of the retina [1]. It is the most common inherited retinal dystrophy, with a worldwide prevalence of 1:4000 [2]. Patients with RP commonly present with night blindness, followed by progressive loss of peripheral vision and subsequently central vision loss [3]. The fundus in RP is characterized by bony spicules, attenuated vessels, and waxy pallor of the optic nerve [4].

This group of retinal diseases may be inherited as autosomal dominant (AD), autosomal recessive (AR), or X-linked (XL). Notably, the autosomal dominant form accounts for 15-25% of cases, while the autosomal recessive and X-linked forms represent 5-20% and 5-15%, respectively [5].

Cataracts are a secondary cause of vision impairment in patients with RP. The most common morphology is posterior subcapsular cataract. In a study done by Pruett in 1983, cataracts were found in 46.4% of the eyes in 192 patients with RP. Out of those, 93.6% showed posterior subcapsular opacification [6]. Previous studies have indicated that the intraocular microenvironment can undergo alterations during the inflammatory response of RP, potentially contributing to the disease's pathogenesis and increasing the risk of cataract formation and complications during surgery. [7].

Cataract surgery significantly improves central visual acuity in patients with RP. Improvement is directly proportional to more severe vision impairment due to cataracts [8].

The incidence of cystoid macular edema (CME) in patients with RP has been reported to occur in 10-50% of patients [9]. CME can lead to a loss of central vision. The pathogenesis of CME in RP patients is poorly understood; however, treatments are available. In a non-randomized controlled clinical study by Huang and coworkers [10], patients with RP and CME who were treated with carbonic anhydrase inhibitors showed better outcomes. Oral acetazolamide should be a first-line treatment in CME secondary to RP [9].

Previous studies have reported similarities in genetic variants and pathophysiology between RP and open-angle glaucoma (OAG). To investigate a further connection, Hung and Chen conducted a comprehensive study using Taiwan's National Health Insurance Research Database from 2001 to 2013 [11]. They enrolled 6,223 patients with RP and 24,892 age- and gender-matched individuals without RP. A Cox regression analysis showed that the RP group exhibited a significantly higher cumulative incidence of OAG (1.57%) compared to the comparison group (0.58%, p < 0.0001). Univariate Cox regression analysis showed a substantial increase in the hazard of OAG development in the RP group, with an unadjusted hazard ratio (HR) of 2.86 (95% confidence interval (CI), 2.21-3.70). Even after adjusting for confounders, the heightened risk persisted (adjusted HR = 2.86; 95% CI, 2.21-3.70) [11]. This nationwide population-based cohort study underscores a significant association, indicating that individuals with RP face a notably elevated risk of developing OAG compared to those without this retinal disease.

Studies by Fernández-Martínez and coworkers suggest that RP patients with variants of *RPGRIP1* may develop OAG [12]. We report on three patients with X-linked retinitis pigmentosa (XLRP) who developed OAG. One patient following cataract surgery and two patients following intraocular steroid injection for the treatment of macular edema as part of RP.

## Case presentation

Case 1

A 17-year-old male patient underwent a thorough ophthalmic assessment conducted by at least one of the authors (Izquierdo N). The best-corrected visual acuity was 20/50-2 in the right eye and 20/50+1 in the left eye. Refraction was +2.00 +1.00 x 90 and +1.00 +2.25 x 90 in the right and left eye, respectively. Upon fundus examination, the patient had cupped optic nerves with an average cup-to-disc ratio of 0.16 right eye (OD) and 0.06 left eye (OS). The patient had arteriolar attenuation with mid-peripheral bony spicules.

Upon optic nerve coherence tomography (Carl Zeiss Meditec, Inc.), the patient had a retinal fiber layer of 143 mm OD and 126 mm OS.

**Figure 1:**
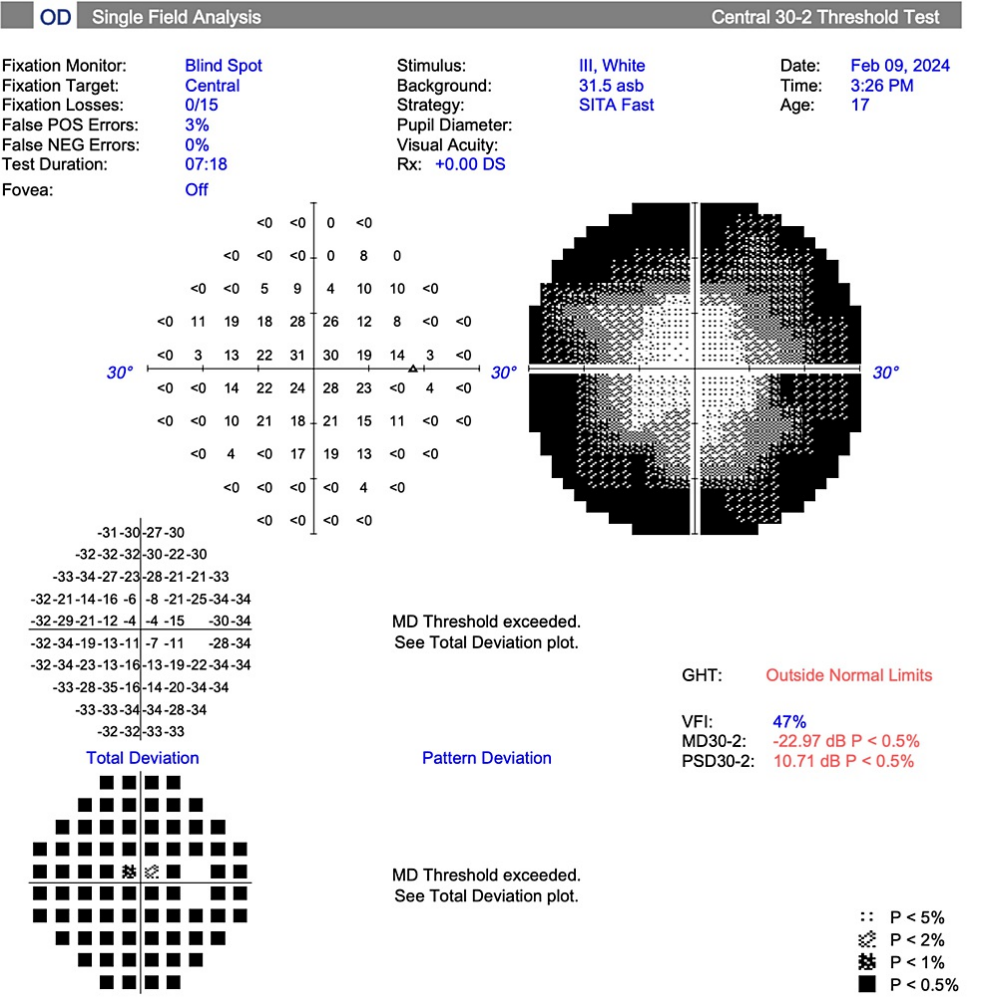
Visual field test results, right eye (Case 1)

**Figure 2:**
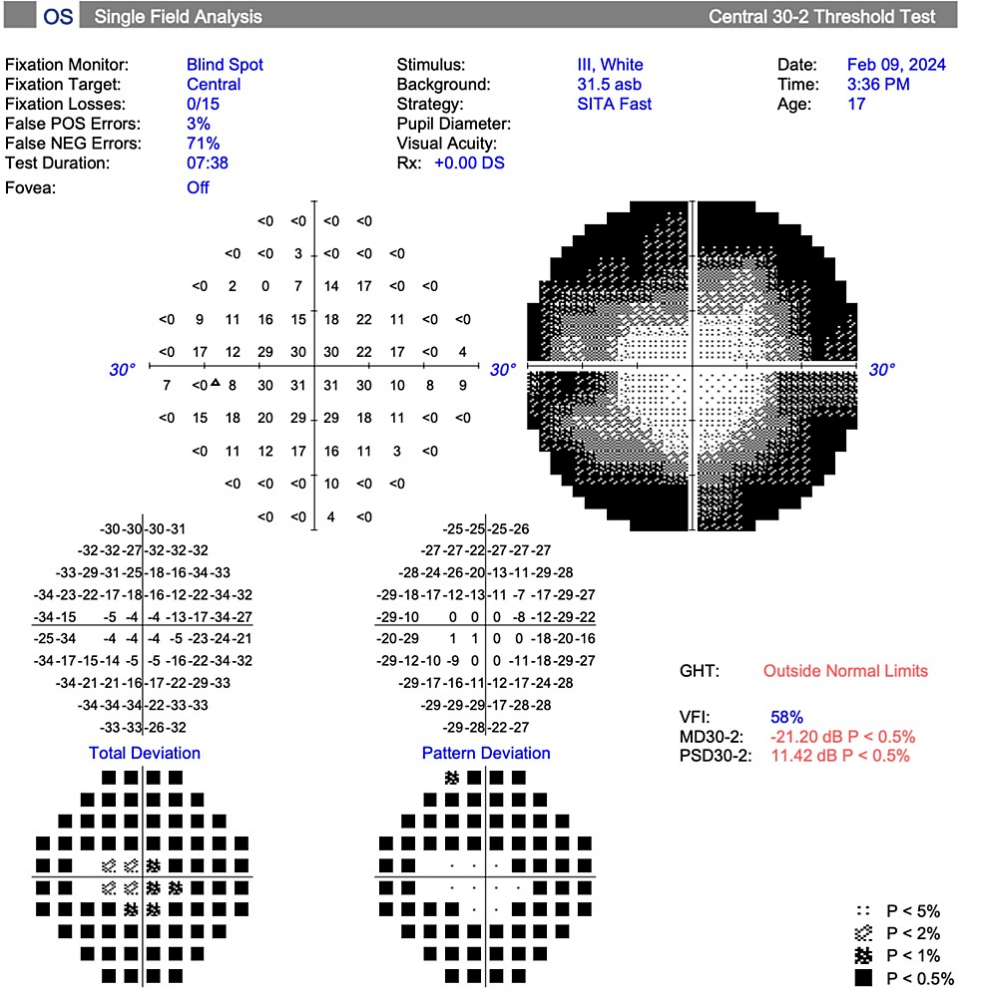
Visual field test results, left eye (Case 1)

Visual field testing (30-2 Carl Zeiss Meditec, Inc.) revealed a mean deviation of -21.71 dB (p<0.5%) OD and -20.50 dB (p<0.5%) OS (Figures 1, 2).

The patient underwent cataract surgery with intraocular lens implantation. The intraocular pressure was 30 OD and 25 OS. The slit lamp examination was unremarkable except for a posterior chamber intraocular lens in both eyes.

Case 2

A 15-year-old man underwent a thorough ophthalmic examination performed by one of the authors (Izquierdo N). The best-corrected visual acuity was hand motion (HM) and 20/40-2 in the right and left eye, respectively. Refraction was +2.00 +1.00 x 90 and +3.00 +1.50 x 90 in the right and left eye, respectively. The intraocular pressure was 31 OD and 28 OS. The slit lamp examination was unremarkable except for a posterior chamber intraocular lens in both eyes. Upon fundus examination, the patient had cupped optic nerves with an average cup-to-disc ratio of 0.45 and 0.49 in the right and left eye, respectively.

Upon optic nerve coherence tomography (Carl Zeiss Meditec, Inc.), the patient had a retinal fiber layer of 209 µm OD and 150 µm OS. Retinal nerve fiber layers were asymmetric (66%).

**Figure 3:**
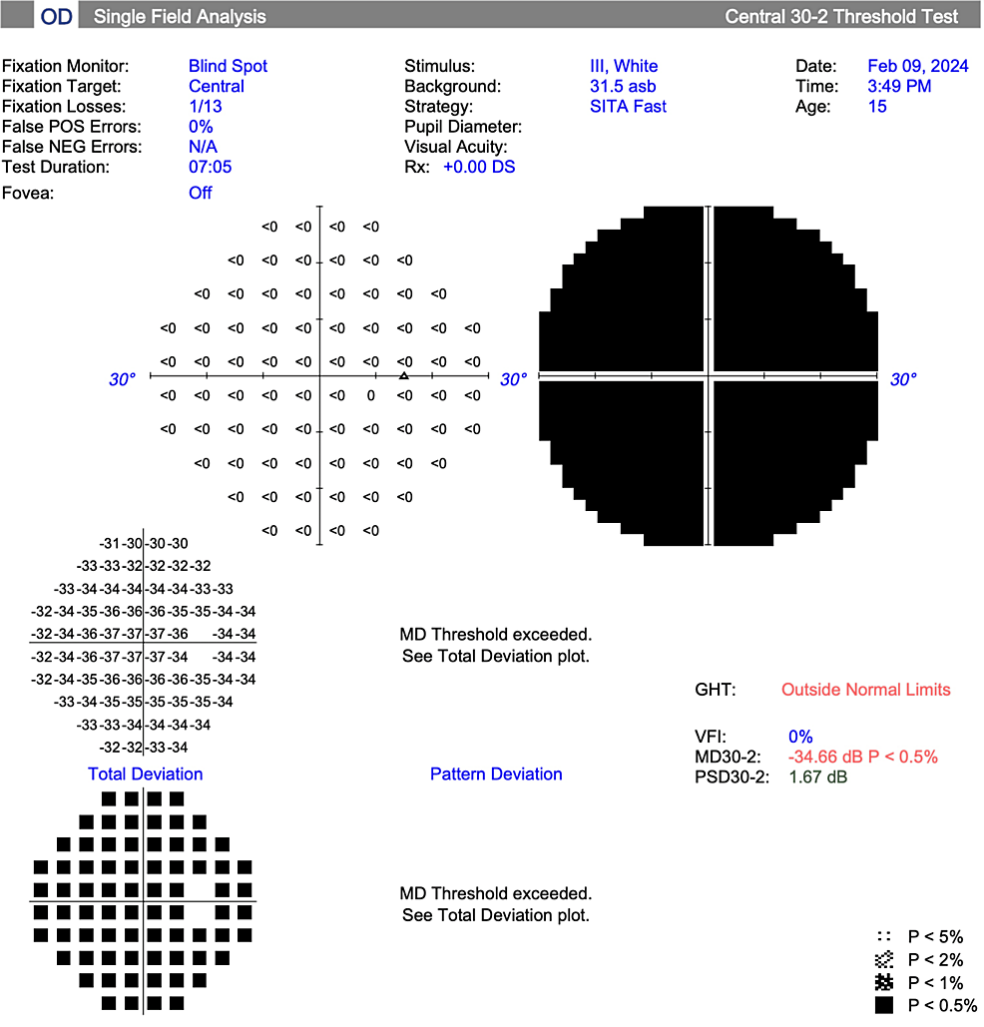
Visual field test results, right eye (Case 2)

**Figure 4:**
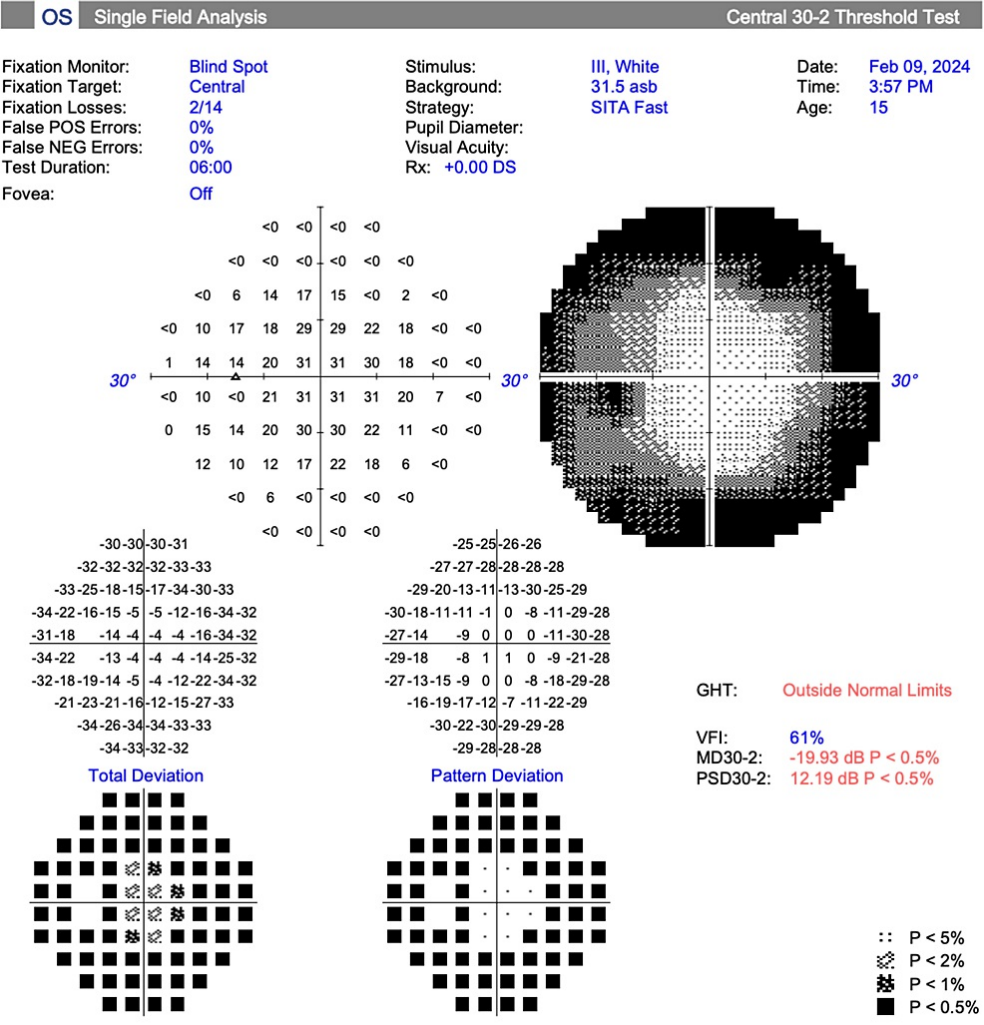
Visual field test results, left eye (Case 2)

Visual field testing (30-2 Carl Zeiss Meditec, Inc.) showed a mean deviation of -34.30 dB (p<0.5%) and -19.56 dB (p<0.5%) in the right and left eye, respectively (Figures 3, 4).

The patient underwent cataract surgery with intraocular lens implantation. The patient underwent an Ahmed valve implantation in the right eye.

Case 3

**Figure 5:**
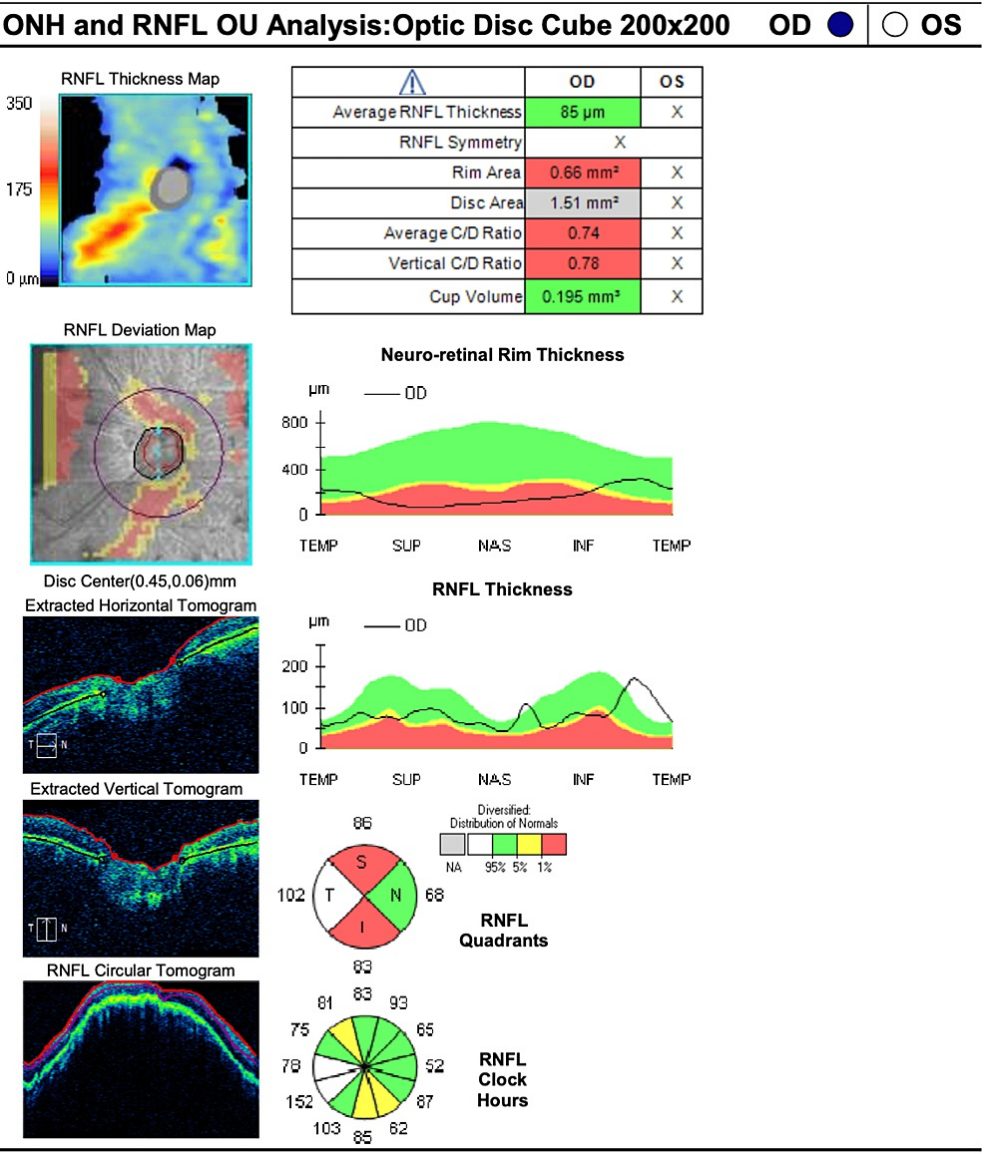
Optic nerve tomography, right eye (Case 3) ONH: optic nerve hypoplasia; RNFL: retinal nerve fiber layer

A 53-year-old female patient underwent a comprehensive ophthalmic evaluation by at least one of the authors (Izquierdo N). The best-corrected visual acuity was HM and no light perception (NLP) in the right and left eye, respectively. Pre-operative refraction was -15.00 +4.00 X 95 and -14.50 +3.50 X 100 in the right and left eye, respectively. The intraocular pressure was 12 OD and 11 OS. The slit lamp examination showed posterior subcapsular cataracts in both eyes. Upon fundus examination, the patient presented with pale optic nerves (an average cup-to-disk ratio of 0.74 OD (Carl Zeiss Meditec, Inc.)), arteriolar attenuation, and bony spicules (Figure 5). Visual field testing (30-2 Carl Zeiss Meditec, Inc.) was not done due to poor vision. 

## Discussion

Several comorbidities have been found in patients with RP, including cataracts, glaucoma, and macular edema. They may all lead to decreased visual acuity.

Macular edema has been reported in patients with RP. Two of the cases developed macular edema as part of the RP. Up to 50% of patients with RP may develop macular edema [9]. Eksioglu and coworkers have reported that patients with RP may develop increased intraocular pressure following the use of steroids for the treatment of macular edema [13]. Patients one and two developed high intraocular pressure and steroid-induced glaucoma following intraocular steroid injections for the treatment of macular edema as part of retinitis pigmentosa. These patients had a mutation (c. 1540del, p.Leu514Trpfs*61) in the *PDE6B* gene.

Studies by Strong and coworkers have reported that there are several therapies for macular edema in patients with RP, including intravitreal steroids [14]. Two of our patients developed high intraocular pressure following an intravitreal steroid injection. Findings in our patients suggest that patients with RP associated with mutations in the *PDE6B* gene may become steroid responders.

Previous studies have reported that an Ahmed valve implantation is an alternative and safe option in the management of resistant, elevated intraocular pressure (IOP) secondary to steroid treatment for macular edema in patients with RP [13]. One of our patients underwent an Ahmed valve implantation for the treatment of a resistant, elevated IOP.

Open-angle glaucoma has been reported in patients with RP following cataract surgery [7,8]. Fernández-Martínez and coworkers reported patients with RP due to variants in the *RPGRIP1* gene who developed open-angle glaucoma [12]. Our third patient had a mutation in the *RPGR* gene (c.2270_2271del, p.Glu757Glyfs*12). One of our patients developed high intraocular pressure following cataract surgery. She had a mutation in the *RPGR* gene. This finding is compatible with previous literature [11]. Females who are carriers of the mutation exhibit a wide range of fundus appearances, spanning from normal to significant retinal degeneration. Rozet and coworkers reported that the age at onset of disease in affected female patients was delayed when compared to male patients with similar variants. In general, retinal disease tends to be less severe in female carriers of XLRP compared to male patients [15].

More than 70% of individuals diagnosed with XLRP have variants in the *RPGR *gene [16]. Nonetheless, *RPGR* variants have also been identified in patients with different types of retinal dystrophies, such as cone-rod dystrophy, atrophic macular degeneration, and syndromic retinal dystrophy accompanied by ciliary dyskinesia and hearing loss, particularly those involving variants affecting exon 6 of the *RPGR* gene [17].

Based on the currently available evidence, the *RPGR* (c.2770_2271del, p.Glu757Glyfs*12) mutation is classified as pathogenic due to its disease-causing role [15].

A population-based case-control study conducted in Taiwan showed a significant association between RP and angle-closure glaucoma (ACG). The study demonstrated that patients with RP had a substantial 3.64-fold increase in the odds of developing ACG compared to the general population [18]. In our study, none of the patients had angle-closure glaucoma.

Matsuo examined photoreceptor outer segments and inflammatory cells found in the fluid aspirated from the eyes of patients diagnosed with Schwartz syndrome. He proposed that these outer segments of photoreceptors might traverse the retinal break and enter the pathways through which aqueous humor drains, potentially causing blockages in outflow. In cases of Schwartz-Matsuo syndrome, the intraocular pressure (IOP) usually reverts to normal levels after surgical repair of a detached retina. We propose that patients with RP who undergo cataract surgery may have increased intraocular pressure due to an outflow disturbance like the one occurring in the Schwartz syndrome [19,20]. 

This case series unveils a complex association between RP and key comorbidities in these patients, with a focus on cataracts, glaucoma, and macular edema. Cataract surgery in patients with RP shows a link to the emergence of glaucoma, particularly in those with *RPGR* and *PDE6B* gene mutations, revealing a novel association with *PDE6B* mutations not previously documented. Furthermore, the paper explores a unique parallel with Schwartz-Matsuo syndrome, suggesting that patients with RP undergoing cataract surgery may develop increased intraocular pressure due to an outflow disturbance akin to Schwartz syndrome. This novel perspective deepens our understanding of the pathophysiological mechanisms governing intraocular pressure dynamics in patients with RP.

Limitations of the study include the small sample size. Further studies evaluating increased intraocular pressure and glaucoma, all types included, in patients with RP are warranted.

## Conclusions

To our knowledge, this is the first report of steroid-induced glaucoma in patients with RP due to mutations in the *PDE6B* gene. Intraocular pressure evaluation remains of utmost importance in the follow-up of patients with the disease.
